# Commentaries on *the Organon**Read before the Homœopathic Union

**Published:** 1894-02

**Authors:** B. Fincke

**Affiliations:** Brooklyn, N. Y.


### COMMENTARIES ON *THE ORGANON.**

Provings on the healthy with high potencies.—*Organon,* §§ 128–137.

B. Fincke, M. D., Brooklyn, N. Y.

Under the auspices of Dr. Ad. H. Gerstel, in Vienna, in 1844, provings of Aconitum Napellus were made by sixteen provers which in boldness exceeded anything of the kind known before.

Dr. Arneth took 100 drops of tinctura fortis in nine days, and after taking the 3d, 2d, and 1st dilution in succession by the tablespoonful for four days without sufficient effect, resumed the strong tincture after ten days, and took 370 drops in three days, when he could not obtain any more symptoms.

Dr. Gerstel took 233 drops tinct. fort. in fifty-five days with good results. He showed little receptivity to larger doses, which affected him more, but produced nothing new.

His nursing wife took 6 drops in 5 ounces of water, which produced some fever-symptoms, but the nursling was not affected. A second dose of 20 drops tinct. fort. brought out a vesicular eruption.

His children, three to seven years of age, took 3–6 drops tinct. f. without effect.

Dr. Maschauer took 545 drops t. f. in twenty-two days. After 10 drops the first two days there was no action at all.

A medical student, twenty-three years, took 2,386 drops t. f. in sixty days with great effect.

Dr. Reisinger took 1,293 drops t. f. in ninety-four days. His rheumatic affections appeared on the days when he took no medicine, and disappeared on resuming it.

---

*Read before the Homœopathic Union.

Dr. Rothansel took 105 drops t. f. in daily increasing doses, by 1 drop, in ten days. He observed that wine, instead of being an antidote, as Hahnemann says, increased the action.

Dr. Schwarz took 1,669 drops t. f. in sixty-nine days with great effect.

Dr. Sterz took 285 drops t. f. in eighteen days; then 100 drops 1st cent. one day; the next five days 100 drops of the 2d cent., rising every day with the potencies to the 3d, 4th, and 5th, until the sixth day he took 100 drops of the 6th cent. A week later, through three days again, 100 drops of the 1st cent. The symptoms from all these low dilutions were of little account.

Dr. Watzke took 1 drop t. f., rising every day by 1 drop for ten days, without regard to diet, taking wine, beer, coffee, etc., and found that they were not antidotes to Aconite.

For a second experiment he took 15 drops t. f.; for the third, 80 drops t. f.; for the fourth, 400 drops t. f. in eight days. Two months later he took 40 drops of the 12th cent. and 10 drops of the same the next day without effect. The day after he took 10 drops of the 8th cent., which repeated some previous symptoms.

This is the same Dr. Watzke who, after all the laborious provings of large doses of crude tinctures, confessed that in spite of his own distrust of the higher Hahnemannian potencies, all the symptoms of Hahnemann obtained by the 30th cent. of Natrum-muriaticum had not only been confirmed, but that the Hahnemannian observations were even more valuable.

Dr. Wurm took 2,325 drops t. f. in fifty days, with a specially meagre result. This prover was plethoric, of lively character, bilio-nervous or sanguine constitution, dark hair, vivid color of the face, and therefore, according to Noack & Trink's *Materia Medica*, should have been peculiarly susceptible to the action of Aconite, which he was not. Thus much for generalization of medicinal action!

But the palm of these provings must be rendered to Dr. and Professor Joseph von Zlatarovich, who took no less than 5,000 drops of the strong tincture in sixty-eight days, and then 1,010

drops of the 2d cent. in sixteen days, and furnished a considerable array of symptoms.

Dr. Gerstel, at the close of his remarkable report, regrets "that many of our colleagues, though animated by the best intention, could not disabuse themselves of the idea that they must be thoroughly impregnated with the medicine, taken uninterruptedly and continued mostly in increasing doses, so that they do not only disturb the action of a pretty well adapted dose, by the following doses, but also produce a merely chaotic picture of the medicinal disease which is of little value."

This is exactly what Hahnemann teaches in the sections on proving which we are just discussing. His warning not to apply large and frequently repeated doses in provings was as little heeded as his advice to give only single infinitesimal doses in treating the sick. What the after-affects upon those provers may have been who took such enormous doses of the crude Aconite-tincture, history does not tell. But it seems almost impossible that they should have escaped ill effects, even if they had not been very susceptible to the medicine. One warning example occurs in the letter of Hahnemann to Stapf d. d. September 16th, 1828, where he writes, "If Franz and the others are not taught by such sad experiments upon themselves considering the necessity of most possibly small doses, I do not know what to think of it. Do I merit such a distrust on what I wrote? Or do they deem themselves more wise?"

Franz in proving Zincum-metallicum had taken the first day 13 grains of the first centesimal trituration; after two months the first day 6 grains, then every sixth and eighth hour 8 grains of the first trituration, and killed himself.

Hering also remarks in his criticism on the Austrian provings that a too great turmoil of symptoms caused by strong doses is always suspicious, just as all symptoms enforced by large doses. With moderate doses there is no such danger. He knew, for he had taken already in 1822 Iodine and Arum in enormous doses; in 1823, Paris and Tartarus-emet.; in 1824, Mezereum and Sabadilla; in 1825, Sabina and Plumbum; in 1826, Cantharides; in 1827, Caladium; in 1828, Lachesis in the first trituration, Mezereum, Sabadilla, Sabina, Cantharides, and Caladium in doses of 10, 20, 50, to 100 drops of the strong tincture for weeks. But he gave it up because the experience taught him that "the coarsest, most violent, and forcible symptoms are the most useless, because every powerful medicine shows them."

You ask whether the heroic provings of the Austrians gave a greater and better harvest than Hahnemann's provings. To answer this question would necessitate a careful comparison of the respective symptoms, for which our time would be too short.

The number of Hahnemann's symptoms of Aconite amounts to 541, that of the Austrians to 712. For the present it must suffice to quote what Dr. Gerstel judges about the provings at the close of his report: "It is remarkable how precisely in expression but still more in essence the communications of our single provers correspond * * * and how nearly all the former observations of Hahnemann are confirmed and explained, so that our proving of Aconite and that of Hahnemann mutually verify each other."

My own first experience dates back to 1849, when I had prepared my first potencies according to Hahnemann up to the 30th potency, at a time when I had not yet seen *The Organon.* A lady friend some sixty years of age, perfectly healthy, satisfied my curiosity as to the efficaciousness of this preparation by taking at ten P. M. one drop of this 30th cent. of Aconite at bed-time.

After half an hour the following symptoms were observed:

Sensation at the back of the head as if the scalp were scraped off toward the top; burning and sensation of dryness in the eyes as after much weeping or from dust in hot weather; sensation as if the eyes were smaller and waved to and fro; burning in the face and drawing in the malar bones; distended and hard abdomen, tender to touch, thicker on the right side, that she could not lie on it; sensation of weakness ascending from the stomach almost to fainting; corns inflamed; feet feel as if swollen; burning in the feet from the soles to the ankles; dullness of the head; sensation in the throat as of mucus obstructing the passage; cool sensation in the nose, extending free and easy into the brain.

These symptoms lasted till twelve o'clock, followed by sound sleep.

In the morning, at the root of the tongue burning.

Though these symptoms are somewhat different from those of Hahnemann, they are clear and defined, and form an addition to what we have.

Since that time, as you well know, we have ascended in the scale of potencies, without reaching the terminus of efficaciousness, and we have found that even the higher potencies are able to produce pathopoëtic pictures not inferior in distinctness and trustworthiness to the provings obtained by the lower potencies and crude drugs, and forming a confirmation, supplement, and complement of the latter. The opposition of the materialists against high potencies cannot alter what has been observed by their advocates, upon competent persons, with as much care and foresight as they claim for themselves. The character of such provings evidences their genuineness by comparison with previous provings. But the materialists reject the high-potency provings altogether, whether they confirm old provings or not, on the ground that no medicinal force can reside in a preparation exceeding the 12th centesimal, in which a stray atom of some substance may have been detected. On the other hand, if a non-sensitive person furnished symptoms with the crude substances and low potencies in large and repeated doses, they are accepted without hesitation, because palpable substances have been applied. The tacit premise is accepted that these symptoms have appeared on account of the materiality of the medicine applied. But Hahnemann has decidedly dismissed that premise, not only on theoretical, but also on practical grounds, for, owing to his primary position, that diseases are the consequence of distunement of the life-force (section 12) they can only be treated by similar dynamical or virtual forces of suitable medicine. Though, in the beginning, proving the medicines in appreciable doses, he, in his progress to better knowledge by experience, applied them in infinitesimal doses, the result of which we have, in the inestimable provings, contained in the work on *Chronic Diseases.* The deniers of the action of high potencies in health and disease, therefore, make themselves guilty of a fallacy, because their argument turns upon the inadvisability of immaterial doses of medicine, which is not the criterion of medicinal action. This can consist in nothing else than experience, observation, and experiment. If all the scientific rules are observed, the result of these irrefutable means of acquiring knowledge of medicinal action must decide it, whether proving and healing with high potencies have a legitimate status in homœopathics or not. The accumulated experience of the homœopathicians answers this question affirmatively.

Therefore, the Hahnemannian advice, given in sections 128–137, deserves the highest consideration and approval, and, accordingly, the method of giving a dose of a higher potency to a healthy, sensitive person, under the necessary rules of conducting provings and allowing it full sway of action, till the system returns to its natural course of life, is the true way to arrive at greater knowledge of the sphere of action of the medicine in the human organism. Whether it be better to begin at the foot of the ladder and ascend gradually higher, or the reverse, is a matter of experiment. But, whatever pure experiment ever will be made in this direction, they can only serve to enrich our *Materia Medica Pura.* Such provings, to make them easier of access in study and more available for practice, ought to be collected in a special periodical work, continuing from year to year, thus forming an ever-increasing treasure of positive symptoms, a *Thesaurus homœopatho-poëticus.*

